# Role of Estrogen Receptor α in Aging and Chronic Disease

**DOI:** 10.20900/agmr20230005

**Published:** 2023-06-06

**Authors:** José V. V. Isola, Sunghwan Ko, Sarah R. Ocañas, Michael B. Stout

**Affiliations:** 1 Aging and Metabolism Research Program, Oklahoma Medical Research Foundation, Oklahoma City, OK 73104, USA; 2 Genes & Human Disease Research Program, Oklahoma Medical Research Foundation, Oklahoma City, OK 73104, USA; 3 Oklahoma City Veterans Affairs Medical Center, Oklahoma City, OK 73104, USA

**Keywords:** 17α-estradiol, hypothalamus, HPG axis, liver, metabolism, neuroinflammation

## Abstract

Estrogen receptor alpha (ERα) plays a crucial role in reproductive function in both sexes. It also mediates cellular responses to estrogens in multiple nonreproductive organ systems, many of which regulate systemic metabolic homeostasis and inflammatory processes in mammals. The loss of estrogens and/or ERα agonism during aging is associated with the emergence of several comorbid conditions, particularly in females undergoing the menopausal transition. Emerging data also suggests that male mammals likely benefit from ERα agonism if done in a way that circumvents feminizing characteristics. This has led us, and others, to speculate that tissue-specific ERα agonism may hold therapeutic potential for curtailing aging and chronic disease burden in males and females that are at high-risk of cancer and/or cardiovascular events with traditional estrogen replacement therapies. In this mini-review, we emphasize the role of ERα in the brain and liver, summarizing recent evidence that indicates these two organs systems mediate the beneficial effects of estrogens on metabolism and inflammation during aging. We also discuss how 17α-estradiol administration elicits health benefits in an ERα-dependent manner, which provides proof-of-concept that ERα may be a druggable target for attenuating aging and age-related disease burden.

## INTRODUCTION

Signaling through estrogen receptor alpha (ERα) is required for normal reproductive function in mammals. ERα also mediates estrogenic cellular responses in a wide range of nonreproductive organ systems, many of which regulate systemic metabolic homeostasis and inflammatory processes that underlie chronic disease onset. For example, specific mutations and polymorphisms in *Esr1*, the gene that encodes ERα, have been associated with greater body mass and adiposity [1,2], in addition to infertility in both sexes [3,4]. Other *Esr1* mutations have been linked to osteoporosis, breast cancer, and Alzheimer’s disease (AD) in females [5,6]. Similarly, the global ablation of ERα in mice increases adiposity and reduces insulin sensitivity in both sexes [7], which further supports the role of ERα in controlling metabolic processes. ERα possesses both genomic (nuclear hormone) and nongenomic (membrane-associated) capabilities [8], which underlies its ability to exert metabolic control in numerous organ systems. It is also noteworthy that numerous *Esr1* splice variants have been identified, several of which are translated into ERα proteins with different molecular weights and functional domains [9]. However, the physiological function of these truncated ERα isoforms remain unknown, particularly with regard to regulating metabolic homeostasis and inflammatory processes. Conversely, many of the truncated ERα isoforms have been associated with tumor cell activity in a variety of cancers [10], which is outside the scope of this mini-review. Despite actions in a variety of metabolically active tissues, the goal of this mini-review is to summarize how ERα modulates metabolism and chronic disease progression through actions in the brain and liver, which we postulate is closely related to the control of systemic aging processes.

## MODULATION OF THE HPG AXIS BY ERα

**Figure 1.**
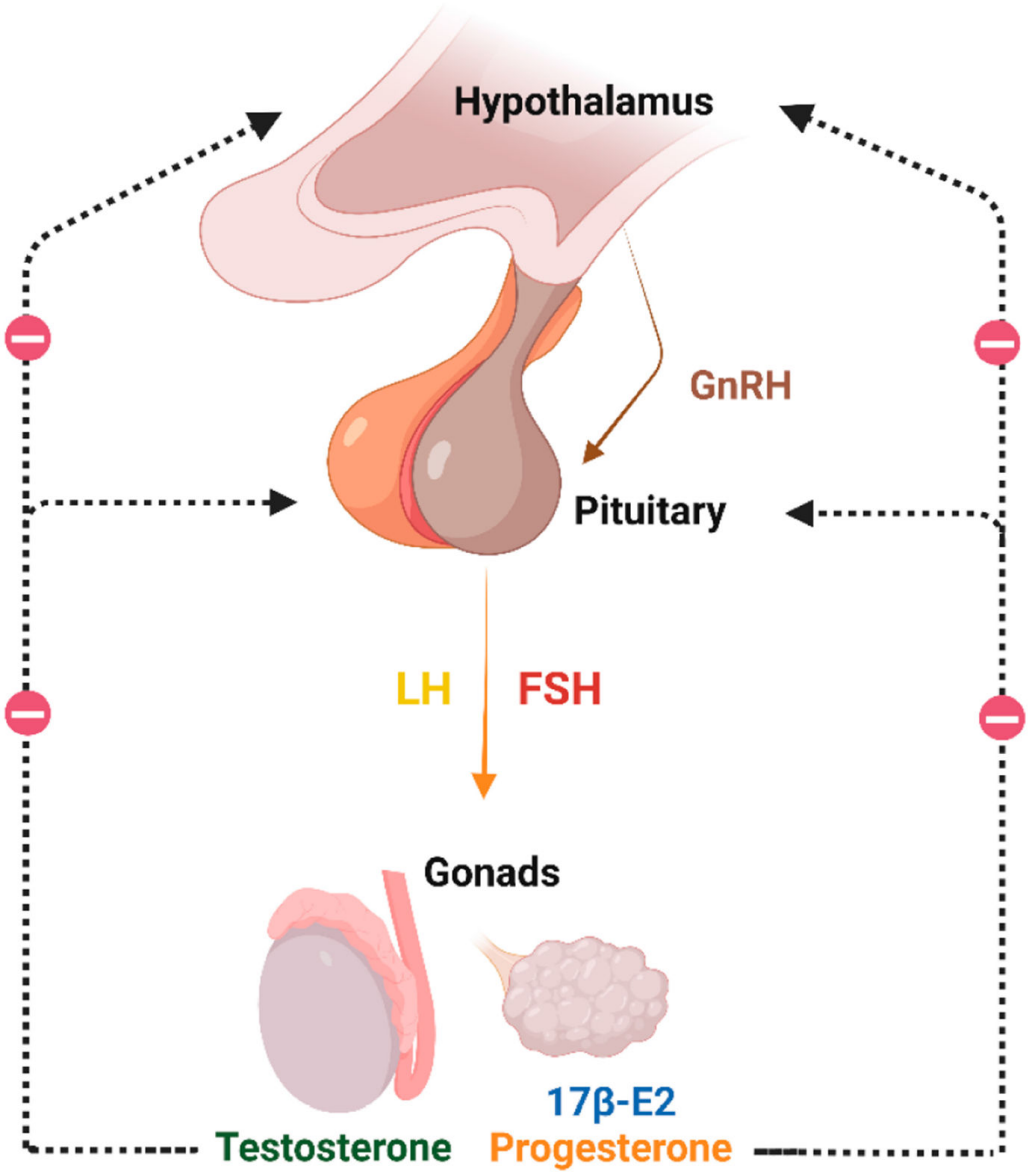
Gonadotropin-releasing hormone (GnRH) is secreted by the hypothalamus and stimulates the production and secretion of follicle-stimulating hormone (FSH) and luteinizing hormone (LH) from the anterior pituitary, which in turn stimulates the production and release of 17β-estradiol (17β-E2), progesterone, and testosterone from the gonads. The release of these hormones controls the production of GnRH and FSH/LH in a negative feedback loop. This figure was created with BioRender.com.

The hypothalamic-pituitary-gonadal (HPG) axis plays a vital role in controlling reproduction, metabolism, and immune function. Gonadotropin-releasing hormone (GnRH) secreted from the hypothalamus serves to stimulate the production and secretion of follicle-stimulating hormone (FSH) and luteinizing hormone (LH) from the anterior pituitary, which in turn stimulates the production and release of sex hormones from the gonads. These sex hormones, predominantly 17β-estradiol (17β-E2) and progesterone in females [11,12] and testosterone in males [13,14], signal in the hypothalamus and pituitary to suppress the production of GnRH and FSH/LH, respectively, as part of the HPG negative feedback loop (Figure 1). 17β-E2 can also signal in males to dampen gonadotropin production, most of which occurs following the aromatization of testosterone to 17β-E2 [15,16]. The aforementioned hormonal cycles regulate germ cell release in females, and germ cell creation in males, therefore the HPG axis is relevant to aging and chronic disease burden because it plays a major role in the established tradeoff effects between reproduction and longevity [17]. With advancing age, gonadal and neuroendocrine changes occur that result in declines in sex hormone production [18], and thus, declines in negative feedback within the HPG axis [19,20]. Age-related hormonal declines are more rapid in females because 17β-E2 production is directly linked to ovarian follicular depletion [21,22]. The reduction in sex hormones leads to elevated production and secretion of GnRH, LH, and FSH, which have been linked to the aging process and a variety of comorbid conditions in sex-specific manners [23-27]. In fact, after menopause, females are confronted with greater risk for numerous age-related diseases with a metabolic and/or proinflammatory underpinning [28-37], several of which rise to incidences commonly observed in males [38].

ERα is the primary receptor involved in 17β-E2-mediated suppression of gonadotropin release in both sexes [39,40], although other receptors, including estrogen receptor beta (ERβ) and G protein-coupled estrogen receptor (GPER), have also been reported to play a role in this process) [41], but are outside the scope of this mini-review. several different neuronal populations have been implicated in the 17β-E2 negative feedback mechanism [11,42,43]. GABAergic neurons in the preoptic area (POA) are believed to provide input to the GnRH negative feedback system [42,44]. There is also evidence that ERα is expressed in a subset of GnRH neurons within the POA, which suggests the possibility of direct regulation of GnRH production by ERα [45]. Further investigation has revealed a functional hierarchy among the various possible mechanisms involved in the HPG feedback process. ERα expression in the arcuate nucleus (ARN) has been demonstrated to be crucial to maintaining reproductive function and E2-dependent negative feedback [46,47]. Although selective knockdown of ERα in kisspeptin neurons within the ARN was found to have no effect on LH secretion [48], these mice exhibited GnRH pulse activity similar to that of gonadectomized mice with high frequency, low amplitude LH pulses [49]. These results suggest 17β-E2 signaling through ERα in kisspeptin neurons in the ARN is the principal mechanism responsible for controlling GnRH pulsatility in mice. Interestingly, ERα in the pituitary has also been implicated in 17β-E2 feedback, and its ablation causes infertility in female mice [50]. Collectively, the findings outlined above indicate that ERα plays a major role in controlling HPG activity, which could conceivably make it a pharmacological target within the hypothalamus and/or pituitary for attenuating aging and chronic disease burden. For instance, agonizing ERα in a manner that curtails age-related increases in GnRH, LH, and FSH production could potentially blunt mechanisms that promote arthritis, kidney disease, obesity, metabolic dysfunction, and neuroinflammation in a sex-specific manner [23-27].

## ROLE OF ERα IN NEUROINFLAMMATION

**Figure 2.**
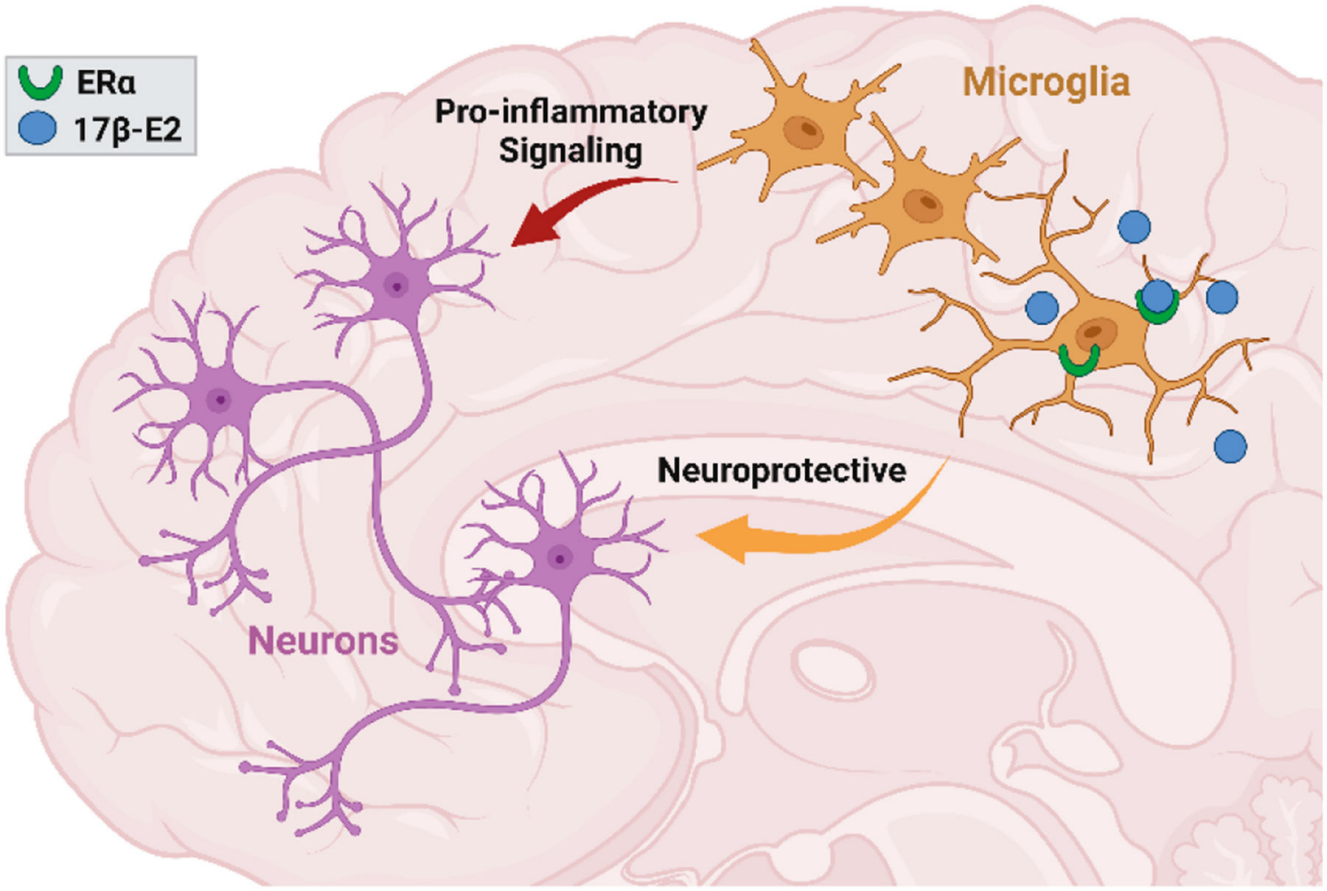
Prior studies show that ERα agonism on microglia, the brain-resident immune cells, limits their transition towards a pro-inflammatory phenotype which has been linked to neuroprotection. This figure was created with BioRender.com.

Estrogens are known to exert anti-inflammatory and neuroprotective effects by agonizing ERα. Interestingly, ERα in microglia have been shown to temper pro-inflammatory processes in both female and male rodents (Figure 2) [51-54]. Microglia are brain-resident immune cells that serve diverse functions across the lifespan, including debris clearance, synaptic pruning, and response to infectious agents [55-57]. In vivo and in vitro studies show that ERα agonism limits the transition of microglia toward pro-inflammatory phenotypes when challenged with noxious stimuli such as bacteria [58] and viruses [59]. Furthermore, synthetic ligands for ERα have also been shown to attenuate the production of tumor necrosis factor α (TNFα), interleukin-1β (IL-1β), and macrophage inflammation protein-2 (MIP2) in primary microglial cultures [60].

Interestingly, ovariectomy (OVX) increases a large number of markers associated with microglial reactivity, including the recognition of inflammatory stimuli and phagocytosis in female rodents [61]. The administration of 17β-E2 in the setting of OVX prevents microglia phenotypic switching, suggesting that ERα agonism plays a critical role in regulating microglia homeostasis [53,61-63]. ERα density within the mouse hippocampus is dramatically reduced with advancing age in female mice [64,65], suggesting that ERα in the aging brain is associated with impaired anti-inflammatory activity and microglial-mediated neurotoxicity. In support of this, global ERα knockout (ERαKO) mice display increased hippocampal expression of IL-1β, interleukin-6 (IL-6), and interleukin-12p40 (IL-12p40), all of which are linked to neurotoxicity [66]. Conversely, chronic treatment with 17β-E2 or selective estrogen receptor modulators (SERMs) in OVX females significantly reduces the number of microglial within the hippocampus [64,67]. This further implicates ERα agonism in neuroprotection during aging and disease processes. Similarly, ERα agonism suppresses microglial neuroinflammation in traumatic brain injury (TBI)-induced male mice by attenuating the decrease in neuronal ERα expression in the ischemic cortex [68]. It should be noted, however, that females generally display a greater prevalence of neurodegenerative diseases, such as AD, and are burdened with more severe pathology and greater cognitive declines than their male counterparts, which worsens following menopause [69,70]. Although definitive mechanisms underlying the aforementioned observations remain unresolved, some reports suggest that declines in ERα agonism during the menopausal transition is a major contributor to female-dominant cognitive declines [71-73], which is further supported by the fact that females receiving estrogen replacement therapies have decreased risk for onset and/or development of AD [74]. If this is indeed proven to be the case, it suggests that ERα plays a greater role in modulating female brain diseases than it does in males, which provides support for the overall goal of developing ERα agonists for treating disease burden in a sex-specific manner. An interesting caveat to the aforementioned findings is the discovery that the maintenance of hippocampal ERα expression, even in the absence of estrogen signaling, is associated with improved cognition in rodents [75,76]. Emerging evidence suggests that ligand-independent activation of ERα, potentially by insulin-like growth factor 1, can affect the transcriptional activity of ERα in a way that improves memory [77]; thereby suggesting that just maintaining ERα expression may be at least partially beneficial for neurocognitive declines.

The molecular mechanisms responsible for ERα-mediated anti-inflammatory effects in the aging brain remain unclear [55]. One potential mechanism is the ERα-mediated regulation of Toll-like receptor (TLR) signaling in myeloid-lineage cells, which has been linked to reduced inflammatory responses [78,79]. Murine and human studies demonstrate that activation of ERα inhibits TLR4 signaling in macrophages and reduces inflammation [80-82]. In addition, ERα interactions with the phosphatidylinositol 3-kinase (PI3K) p85 subunit and AP-1 promoter sites may be involved in blocking TLR4 signaling in macrophages [55]. Another potential mechanism by which ERα agonism may attenuate inflammatory cytokine production following TLR activation is through the inhibition of NF-kB, which has been shown to occur through direct and indirect mechanisms [83,84]. Recent reports have also proposed that 17β-E2 regulates the transition of macrophages into different activation states in an ERα-dependent manner [55]. For example, quantification of inflammatory cytokine production during time-lapse microscopy demonstrated that 17β-E2 inhibits IL-1β and increases interleukin-10 (IL-10) expression, the latter of which is an anti-inflammatory cytokine, during acute lipopolysaccharide exposure [85]. These effects were mediated by suppressor of cytokine signaling 3 (SOCS3), a transcription factor that is partially regulated by ERα, which provided the ability of macrophages to terminate the pro-inflammatory phase [55,86]. Collectively, ERα agonism facilitates intrinsic and extrinsic macrophage programming that allows for the resolution of inflammation. It should be noted that ERα actions in astrocytes have also been shown to provide neuroprotective effects in the brain [87], however discussion of these actions is beyond the scope of this review.

In addition to the role of ERα in modulating chronic brain inflammation, neuronal injury, and neurodegeneration through actions in the hippocampus, amygdala, and cortex [54,88-90], it also plays a major role in regulating pro-inflammatory processes in the hypothalamus [91]. The hypothalamus is one of the most important brain regions involved in the control of feeding behavior, energy expenditure, and systemic glucose homeostasis in both sexes [92]. In the setting of obesity and advancing age, microglia activation is commonly observed in the hypothalamus [93,94], which has been linked to neuronal endoplasmic reticulum stress, declines in insulin and leptin sensitivity, and faster aging in male and female mice [93,95,96]. These events promote hyperphagia and the diminished control of hepatic gluconeogenesis [97], which further exacerbates metabolic dysfunction and the aging process. ERα activity in the hypothalamus has been linked to the aforementioned decline in metabolic function and mechanisms that promote aging, which occurs through actions on both microglia and neurons [53,91,98]. These observations provide additional support for the idea that tissue-specific ERα agonism may serve as a target for delaying the aging process and chronic disease onset.

## ROLE OF ERα IN METABOLIC PLASTICITY

**Figure 3.**
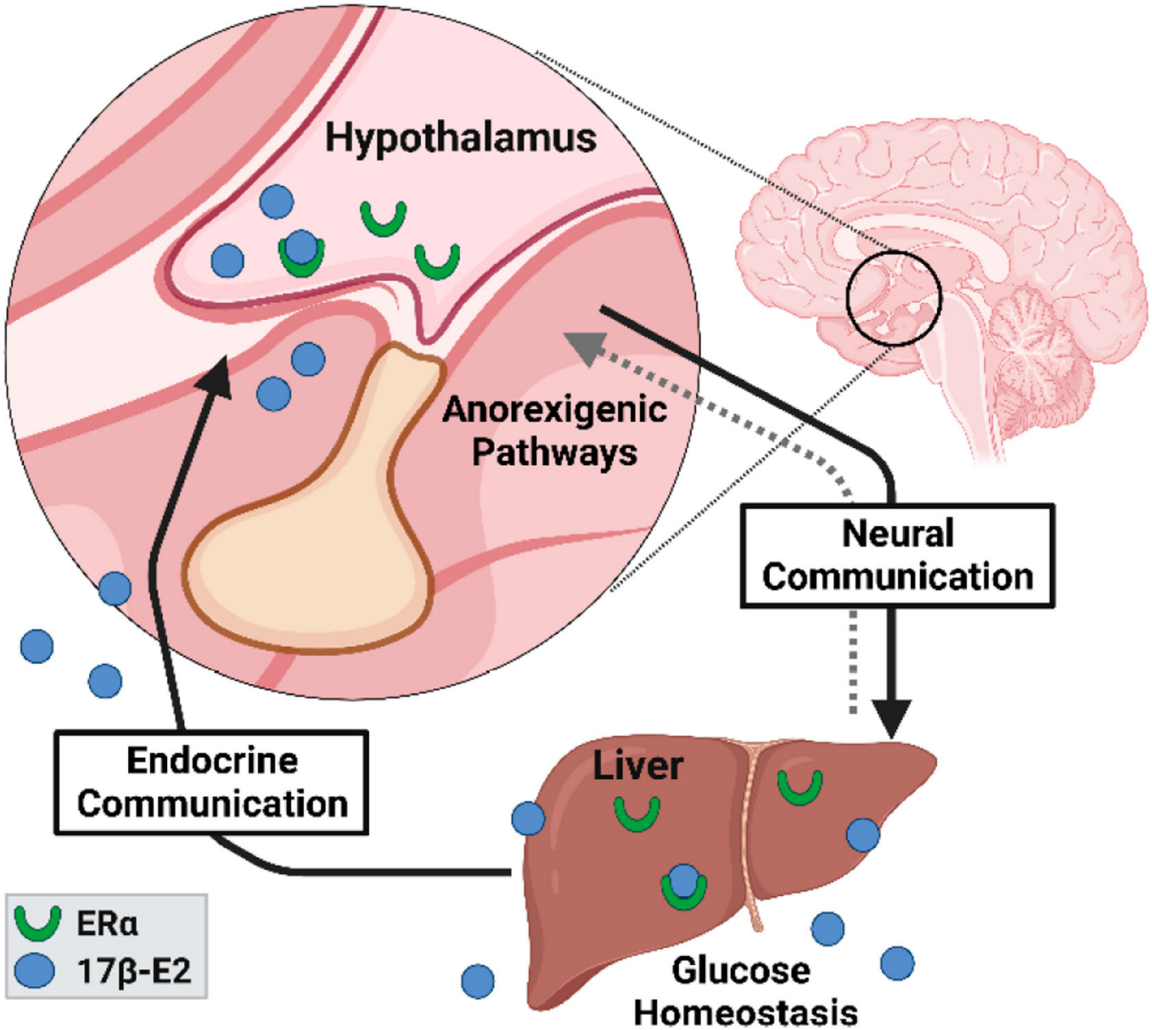
17β-E2 acts through ERα in brain and liver to regulate systemic metabolism by controlling feeding neurocircuitry and macronutrient utilization. All the communication pathways between the two organ systems are still being elucidated. This figure was created with BioRender.com.

There is abundant data demonstrating that ERα is a major regulator of systemic metabolic parameters through actions in the brain and liver [99-101]. ERα has also been implicated in the control of skeletal muscle metabolism by regulating mitochondrial function and quality [102], but this is outside the scope of the current mini-review. 17β-E2 acts through ERα in brain and/or liver to regulate glucose homeostasis, lipid distribution, thermogenesis, and hypothalamic anorexigenic pathways (Figure 3) [57,99,103]. The loss of endogenous estrogen actions after menopause in humans or OVX in mice eliminates these beneficial effects and elicits metabolic perturbations [104] that are nearly identical to those seen in global ERαKO mice [7,105]. Estrogen replacement therapies in both humans and mice reverses the adverse metabolic effects associated with menopause [106,107] and OVX [108]. Most of the prior studies that have evaluated the effects of ERα on metabolic readouts have been done in female mammals, although more recent work has demonstrated that ERα also plays a critical role in modulating metabolism in male mammals. For example, Allard et al. recently reported that genomic actions of ERα regulate systemic glucose homeostasis in mice of both sexes and insulin production and release in males [109]. Other reports have also shown that hepatic steatosis, insulin sensitivity, and the control of hepatic gluconeogenesis are regulated through FOXO1 in an ERα-dependent manner in male mice [110]. Lastly, ERα ablation in hepatocytes abrogates similar estrogen-mediated metabolic benefits [111-113]. Interestingly, ligand-independent activation of ERα in human hepatocytes has been reported to modulate the expression of several cytochrome P450 genes [114], although the role this may play in modulating systemic physiological parameters remains unknown.

In the brain, a variety of hypothalamic neuronal populations are critically important for central control of feeding and energy expenditure. Prior work has shown that brain-specific ERα ablation promotes obesity in both female and male mice [115]. This observation was associated with increased food intake and decreased locomotion and energy expenditure [115]. Other studies employing mice with conditional deletion of ERα indicate that 17β-E2 actions in subsets of pro-opiomelanocortin (Pomc) and agouti-related protein/ neuropeptide Y (AgRP/NPY) neurons within the ARN play critical roles in controlling feeding behavior and energy balance [115-118]. Pomc and AgRP/NPY neurons in the ARN receive and integrate hormonal (e.g., insulin, ghrelin, leptin, cholecystokinin) and nutritional (e.g., glucose, fatty acids) signals from the peripheral circulation as well as neural signals in an effort to coordinate counterregulatory metabolic responses [92]. As mentioned above, obesity and aging are associated with impaired insulin-sensitivity, leptin-sensitivity, and nutrient-sensing in neurons within the ARC, which promotes increased food intake, hepatic gluconeogenesis, and adipocyte lipolysis [92,119]. Interestingly, 17β-E2 signaling through ERα reverses these declines and restores metabolic flexibility through what is currently believed to be direct interactions with insulin and/or leptin receptor signaling in Pomc and AgRP/NPY neurons [120-122]. Pomc- and AgRP/NPY-mediated control of hepatic gluconeogenesis is known to be regulated by sympathetic outflow to the liver [119,123-128], although recent reports suggest that brain-liver crosstalk is almost certainly a bidirectional pathway that is also controlled by nutrient-sensing within the gastrointestinal tract [129,130].

The role that ERα plays in regulating the gut-brain-liver axis remains unresolved, although the ablation of ERα in hepatocytes has been reported to adversely affect AgRP/NPY neuronal activity within the ARN of female mice [131]. It remains unclear if the change in AgRP/NPY activity in hepatocyte ERαKO mice occurs through vagal afferent signaling from the liver, or a change in metabolic substrates and/or endocrine factors being released from liver that cross the blood-brain barrier (BBB) and signal in the ARN. However, the authors did report that hypothalamic microglia in hepatocyte ERαKO mice present morphology indicative of an overt inflammatory phenotype [131], which led to speculation that changes in hepatic lipid metabolism with ERα ablation promotes the production and secretion of pro-inflammatory lipid moieties that cross the BBB and signal in the ARN. Additional studies will be needed to clearly define how hepatic ERα modulates Pomc and/or AgRP/NPY neuronal activity, but an emerging body of literature indicates that 17β-E2, likely through ERα, beneficially modulates vagal afferent signaling in the gut-brain-liver axis [132-135]; highlighting this pathway as a potential therapeutic target for mitigating aging and metabolic diseases.

## HEALTH BENEFITS OF AGONIZING ERα WITH 17α-ESTRADIOL

Although estrogen replacement therapies improve a variety of comorbid conditions and likely elicit benefits on aging processes [136-139], chronic administration has been linked with greater cancer and cardiovascular risks in some female populations [140,141]. Additionally, elevated serum 17β-E2 in males is associated with stroke risk [142], prostate cancer development [143], and feminization [144]. Therefore, the challenge remains of determining how best to exploit the beneficial effects of systemic estrogen therapies while circumventing adverse biological consequences. We and others have begun to address this biological challenge through the use of 17α-estradiol (17α-E2). 17α-E2 is a naturally-occurring diastereomer of 17β-E2 [145,146] that is present in both mammalian sexes [147-149], although circulating levels are quite low. 17α-E2 is also a minor constituent of estrogen replacement therapies [150] but only possesses about 3%–4% of the binding affinity to ERα that 17β-E2 does [151]. 17α-E2 has predominantly been studied as a neuroprotective hormone with mild to moderate efficacy in both male and female models of ischemia, Alzheimer’s, and Parkinson’s diseases [147,150,152-155]. It was not until recently that the effects of 17α-E2 on systemic aging, longevity, and conditions that promote aging (e.g., obesity) were evaluated. The National Institute on Aging Interventions Testing Program has shown that 17α-E2 extends lifespan in male mice when treatment is initiated in mid-life [156,157] and late-life [158]. The magnitude of lifespan extension with 17α-E2 treatment in male mice is similar to that of calorie restriction [159] and rapamycin administration [160], which indicates 17α-E2 elicits potent effects that could conceivably be translated to men.

Our previous work has established that 17α-E2 administration reduces calorie intake and adiposity in conjunction with dramatic improvements in metabolic parameters (e.g., glucose tolerance, insulin sensitivity, ectopic lipid deposition) in obese and/or aged male mice [161-165]. We surmise these benefits underly the lifespan-extending effects of 17α-E2. Others have also reported that 17α-E2 treatment elicits benefits on glucose tolerance, mTORC2 signaling, hepatic urea cycling, markers of neuroinflammation, and sarcopenia [166-170]. Importantly, male-specific benefits occur without overt feminization of sex hormone profiles [161] or reproductive function [171]. Female mice are generally unresponsive to 17α-E2 treatment [166-170,172,173], unless subjected to chronic high-fat feeding over several months (unpublished observation) or following OVX [174]. Until recently the receptor(s) that mediate the actions of 17α-E2 were believed to be uncharacterized [146,148,154,175], although our recent report clearly demonstrated that the majority of health benefits attributed to 17α-E2 treatment are ERα-dependent [163]. This report also established that the hypothalamus and liver are the primary organ systems where 17α-E2 signals to regulate metabolic homeostasis in male rodents. Additional studies are needed to determine if 17α-E2 acts predominantly through ERα in a cell-type-specific manner in the hypothalamus and/or liver to modulate not only systemic metabolic homeostasis, but also aging and longevity. Although not definitive, the data generated thus far indicates that ERα agonism by 17α-E2 in hypothalamic neurons and/or hepatocytes may hold therapeutic potential for attenuating mechanisms that promote aging and chronic disease burden in men.

## FUTURE STUDIES & CONCLUSIONS

The possibility of developing SERMs that modulate ERα for the treatment of aging and age-related diseases in a sex-specific manner is encouraging, but important knowledge gaps remain. Given the widespread expression of ERα isoforms in organs systems throughout the mammalian system, there are opportunities to mechanistically explore the effects of 17α-E2 in these tissues and how they influence metabolism, inflammatory responses, and ultimately aging. However, given the link between ERα activity and cancer in females, rigorous preclinical evaluation of newly developed SERMs and existing ligands, including 17α-E2, is required prior to clinical application. Additional studies that unravel the genomic and nongenomic actions of ERα in the context of metabolic and inflammatory processes are also needed because they could also present opportunities to develop therapies aims at treating sex-specific disease burden. Lastly, differences in ERα regulation between rodents and humans will also need to be carefully considered when attempting to translate newly developed SERMs or 17α-E2 into human studies.

## Data Availability

No new data was generated for the purposes of this mini-review.
